# High mannose level in bladder cancer enhances type 1 fimbria–mediated attachment of uropathogenic *E. coli*

**DOI:** 10.3389/fcimb.2022.968739

**Published:** 2022-08-31

**Authors:** Naseem Maalouf, Chamutal Gur, Vladimir Yutkin, Viviana Scaiewicz, Ofer Mandelboim, Gilad Bachrach

**Affiliations:** ^1^ The Institute of Dental Sciences, The Hebrew University-Hadassah School of Dental Medicine, Jerusalem, Israel; ^2^ Department of Rheumatology, Hadassah Hebrew University Hospital, Jerusalem, Israel; ^3^ Department of Urology, Hadassah Hebrew University Hospital, Jerusalem, Israel; ^4^ The Lautenberg Center of General and Tumor Immunology, The Hebrew University Hadassah Medical School, Institute for Medical Research Israel Canada (IMRIC), Jerusalem, Israel

**Keywords:** UPEC, uropathogenic *E. coli*, bladder cancer, type 1 fimbria, mannose, BCG

## Abstract

Bladder cancer is the 4^th^ leading cancer in men. Tumor resection followed by bladder instillation of Bacillus Calmette-Guérin (BCG) is the primary treatment for high-risk patients with Non-Muscle Invasive Bladder Cancer (NMIBC) to prevent recurrence and progression to muscle-invasive disease. This treatment, however, lacks efficiency and causes severe adverse effects. Mannose residues are expressed on bladder surfaces and their levels were indicated to be higher in bladder cancer. Intravesical instillations of a recombinant *Pseudomonas aeruginosa* (PA) overexpressing the mannose-sensitive hemagglutination fimbriae (PA-MSHA), and of a mannose-specific lectin-drug conjugate showed efficiency against NMIBC in murine models of bladder cancer. Urothelial mannosylation facilitates bladder colonization by Uropathogenic *E. coli* (UPEC) *via* the interaction with the FimH mannose lectin, positioned at the tip of type 1 fimbria. A recombinant BCG strain overexpressing FimH on its outer surface, exhibited higher attachment and internalization to bladder cancer cells and increased effectivity in treating bladder cancer in mice. Investigating the pattern of mannose expression in NMIBC is important for improving treatment. Here, using tissue microarrays containing multiple normal and cancerous bladder samples, and lectins, we confirm that human bladder cancer cells express high mannose levels. Using UPEC mutants lacking or overexpressing type 1 fimbria, we also demonstrate that tumor-induced hypermannosylation increases type 1 fimbria mediated UPEC attachment to human and mouse bladder cancer. Our results provide an explanation for the effectiveness of PA-MSHA and the FimH-overexpressing BCG and support the hypothesis that mannose-targeted therapy holds potential for improving bladder cancer treatment.

## Introduction

Bladder cancer is the fourth most common cancer in men in Europe and the United States (Ferlay et al., 2010; Burger et al., 2013; Nielsen et al., 2014). Men, compared to women, are 3-4 times more likely to develop bladder cancer during their lifetime (Ferlay et al., 2010; Nielsen et al., 2014). Histologically, bladder cancer develops initially in the epithelial layer and mostly remains confined to the mucosa and is defined as non-muscle-invasive bladder cancer (NMIBC). Transurethral resection is the primary standard treatment for NMIBC. Intravesical instillations of live Bacillus Calmette-Guérin (BCG) immunotherapy (Grange et al., 2008; O'Donnell, 2009; Babjuk et al., 2019), or cytotoxic drugs such as mitomycin C, epirubicin and gemcitabine (Chou et al., 2017) usually follow as the main adjuvant therapies for NMIBC. Despite these treatments up to 70% of NMIBC recur after treatment, of which 10% to 20% progress to muscle-invasive bladder cancer (Kaufman et al., 2009).

BCG treatment prevents bladder cancer growth by triggering an immune response against both bacteria and cancer cells (Yutkin et al., 2007). However, BCG immunotherapy is ineffective in about 30% of the patients, and causes severe side effects (Kamat et al., 2017) that result in discontinuous treatment in up to 50% of the patients (Miyazaki et al., 2013; Brausi et al., 2014). In some cases, BCG instillation can lead to life-threatening sepsis (Miyazaki et al., 2013; Brausi et al., 2014). Therefore, there is an urgent need for developing safer, more effective, and more tolerable adjuvant therapeutic agents for treating NMIBC.

Glycosylation is a key cellular mechanism regulating several physiological and pathological functions. Aberrant glycosylation found in cancer, plays a role in tumor progression, and is being explored for tumor detection and targeted therapy (Pinho and Reis, 2015; Višnjar et al., 2019; Huo et al., 2022). The glycan composition that coats the apical surface of superficial urothelial cells (umbrella cells), is altered during urothelial cancer transformation. Mannose is known to be expressed in the normal bladder uroepithelium (Poole et al., 2018; Lupo et al., 2021). Interestingly, Concanavalin A (ConA), a mannose-specific lectin, showed increased binding to cancerous urothelium, compared to the normal one (Takayama, 1984; Neal et al., 1987).

Hypermannosylation in bladder cancer was recently supported in a study demonstrating that ConA displays high selectivity towards both human and mouse bladder cancer cell lines, and in an orthotopic mouse model, can be utilized as an effective vehicle to direct intravesical chemotherapy of bladder cancer (epirubicin) (Huo et al., 2022).

In addition to serving as a target for a lectin-drug conjugate (Huo et al., 2022), mannose overabundance in bladder-tumor was also used to direct bladder cancer immunotherapy. Among the recently proposed immunotherapeutic alternatives to BCG, intravesical injections of *Pseudomonas aeruginosa* (PA) expressing dense mannose-sensitive hemagglutination fimbria (PA-MSHA) showed efficacy as therapy for NMIBC in a mouse bladder cancer model (Wang et al., 2021). PA-MSHA can interact and bind to mannosylated glycoproteins found on the cancer cell surfaces (Liu et al., 2010).

FimH is the distal component of type 1 fimbria that is expressed by many uropathogenic *E. coli* (UPEC) strains (Emody et al., 2003; Gur et al., 2013), and plays a critical role in the interactions of UPEC with D-mannose -enriched bladder uroplakin Ia (Zhou et al., 2001). Overexpression of FimH on the outer surface of a recombinant BCG (rBCG-S.FimH) was shown to markedly improve adhesion, expedite internalization into urothelial cells, and improve its immunotherapeutic effect for treating bladder cancer in mice, compared to the wild-type parental BCG (Zhang et al., 2022).

Here, using tissue microarrays containing multiple normal and tumor human bladder tissues, we re-tested whether mannose level is increased in bladder cancer. We show that a higher level of mannose, but not of uroplakin Ia, is detected in bladder cancer, and that the bladder cancer cells, which overexpress mannose, are preferentially bound by UPEC that overexpresses type 1 fimbria.

## Material and methods

### Tissue microarray analysis

Bladder cancer tissue microarrays (TMAs) BL244, BL244a and T124a were purchased from US Biomax Inc. TMAs BL244 and BL244a contain samples of 12 bladder tumors and their matching normal tissues. TMA T124a contains samples of 10 bladder tumors and two normal bladder tissues (all different from those in TMAs BL244 and BL244a). More details regarding the tissue samples are available on official US Biomax website (BL244: https://www.biomax.us/p_BL244_1298628261, BL244a: https://www.biomax.us/p_BL244a_221856716, T124a: https://www.biomax.us/p_T124a_407306661).

### Bacterial strains and growth conditions

UPEC CFT073 mutants for fimbriae expression UPEC Fim ON and UPEC Fim OFF, were used in this study. In UPEC, expression of the type 1 fimbria is regulated at the transcriptional level by a promoter situated on an invertible element, which can exist in one of two different orientations. The orientation of the invertible element that allows the expression of the fimbria is defined as “on”, and the opposite orientation in which no transcription occurs, is defined as “off” (Gunther et al., 2002). UPEC Fim ON is a UPEC CFT073 mutant in which the promoter-containing invertible element is locked in the on orientation resulting with overexpression of the type 1 fimbria (Gunther et al., 2002). UPEC Fim OFF is a UPEC CFT073 mutant in which the invertible element is locked in the off orientation, preventing expression of type 1 fimbria (Gunther et al., 2002). Both strains were kind gifts from Professor H. Mobley. These UPEC strains were transformed with the GFP expressing pCM18 plasmid (Hansen et al., 2001). GFP expressing bacteria were grown in Luria Broth (LB) for 18 hr at 220 rpm and 37°C in the presence of erythromycin (300 μg/mL).

### Detection of Gal-GalNAc, uroplakin Ia and mannose

TMA tissue deparaffinization and hydration was performed as follows: xylene (Gadot-group) twice for 5 min, xylene 1 min, xylene/ethanol (50%/50%) 1 min, 100% ethanol (Gadot-group) twice 10 min, 90% ethanol 2 min, 80% ethanol 2 min, 70% ethanol 2 min and then washed in PBS for 2 min. Slides were then blocked with PBS supplemented with 10% BSA, 10% FBS and 0.5% Triton X-100 for 2 h at room temperature. Next, the slides were incubated overnight at 4°C with 50 μg/ml in PBS of FITC–conjugated Peanut Agglutinin (PNA) (Sigma-Aldrich, cat. No. L7381), anti-uroplakin Ia antibody (Abcam, cat. No. ab95207) or ConA (Sigma-Aldrich, cat. No. C7642) for the detection of Gal-GalNAc, uroplakin Ia and mannose respectively. Next day, the slides were washed three times with PBS for 10 min each, and then incubated with Hoechst 33258 (Sigma-Aldrich, cat. No. 94403) diluted 1:5,000 in PBS for 15 min at room temperature.

### Detection of bacterial attachment

TMA slides went through deparaffinization and hydration as follows: xylene twice for 5 min, xylene 1 min, xylene/ethanol (50%/50%) 1 min, 100% ethanol twice 10 min, 90% ethanol 2 min, 80% ethanol 2 min, 70% ethanol 2 min and then washed in PBS. Next, sections were blocked with TBS (0.05 M Tris-HCl [pH 7.8], 0.1 M NaCl) supplemented with 20% BSA, 20% FBS and 5% Triton X-100 for 6 hours at room temperature, followed by incubation with 5×10^7^ GFP expressing Fim ON per ml blocking solution, overnight at 4°C. Slides were then washed three times with PBS for 10 min each, and incubated with Hoechst 33258 (Sigma-Aldrich, cat. No. 94403) diluted 1:5000 in PBS for 15 min at room temperature.

For competition assay, Fim ON was pre-incubated with 400 mM mannose or 400 mM galactose or 0.001 mM unconjugated ConA (Sigma-Aldrich, cat. No. L7647) in PBS for 30 min prior to incubation with sections.

### Fluorescence microscopy

Pictures were taken using the Nikon Eclipse Ti microscope.

Several cores were found to have undergone distortion, assumingly caused by improper handling of the slides, and were excluded from the analysis.

### Fluorescence quantification

Fluorescence analyses were performed using NIS Elements microscope imaging software version 5.0 (Zaccard et al., 2021). H&E staining of the sections containing tumor and normal samples is available at US Biomax website (links provided above in “Tissue microarray analysis”). For tumor samples, the whole core was determined as region of interest (ROI). For normal bladder samples, only the lumen epithelial area was determined as ROI. The ROI in each sample quantified by the software in mm^2^.

For analysing fluorescence levels of the conjugated lectins, the software calculated the fluorescent intensities that were above the predetermined threshold, and then, these values were divided by the ROI area to get the final ratios (fluorescence intensity/area (mm^2^)).

The bacteria were counted by measuring the area of florescence (higher than the predetermined threshold) in the ROI, divided by mean fluorescence of the area of a single bacteria, which was determined in three arbitrary pictures. The bacterial numbers were divided by the ROI area to get the final ratios (bacteria/mm^2^).

### Cell lines and tissue culture

Mouse bladder cancer cell lines, MBT2 (derived from C3H mouse) and MB49 (derived from C57BL/6 mouse), were cultured with RPMI with 10% fetal bovine serum, 1% L-Glutamine, penicillin–streptomycin and amino acids (Biological Industries).

### Mannose quantification using flow cytometry

FITC-labeled ConA (Sigma-Aldrich, cat. No. C7642) was incubated at a final concentration of 160 nM (in PBS) per 2.5x10^5^ MB49 cells in 96 well plates, for 30 min at room temperature and gentle shaking. Cells were then washed twice with PBS, prior to flow cytometry (Accuri, C6 flow cytometer, BD, USA). For competition experiments, mannose or galactose was added to wells, to a final concentration of 100mM or 400mM. Data analyses were performed using Flowjo 10.0.8 software (Tree Star, Ashland, OR, USA).

### Quantification of bacterial attachment to bladder cancer cell lines using flow cytometry

2.5x10^6^ GFP-expressing UPEC Fim ON or Fim OFF were incubated in PBS with MBT2 or MB49 cells at a multiplicity of infection (MOI) of 10 in 96 well plates, for 3 hours at room temperature and gentle shaking. The cells were then pelleted by centrifugation at 4°C, 1000 rpm, for 5 min and washed twice with PBS prior to flow cytometry (Accuri, C6 flow cytometer, BD, USA). For the competition experiments, mannose (100mM, 400mM), galactose (100mM, 400mM) or unconjugated ConA (80, 160, 320, 640 and 1280 nM) were added together with bacteria and cancer cells.

Flow cytometry analysis were performed using Flowjo 10.0.8 software (Tree Star, Ashland, OR, USA).

### Statistical analysis

GraphPad Prism software (Version 6.0) used for figures graphics and statistical analysis as described in the figure legends. The various groups were compared against each other in matched pairs, we therefore used Wilcoxon test or t-test to calculate *p* values. The multiple comparisons were adjusted *via* Bonferroni corrections. Differences were considered statistically significant at *p* < 0.05.

## Results

### Mannose is highly expressed in human and murine bladder cancer cells

**Figure 1:**
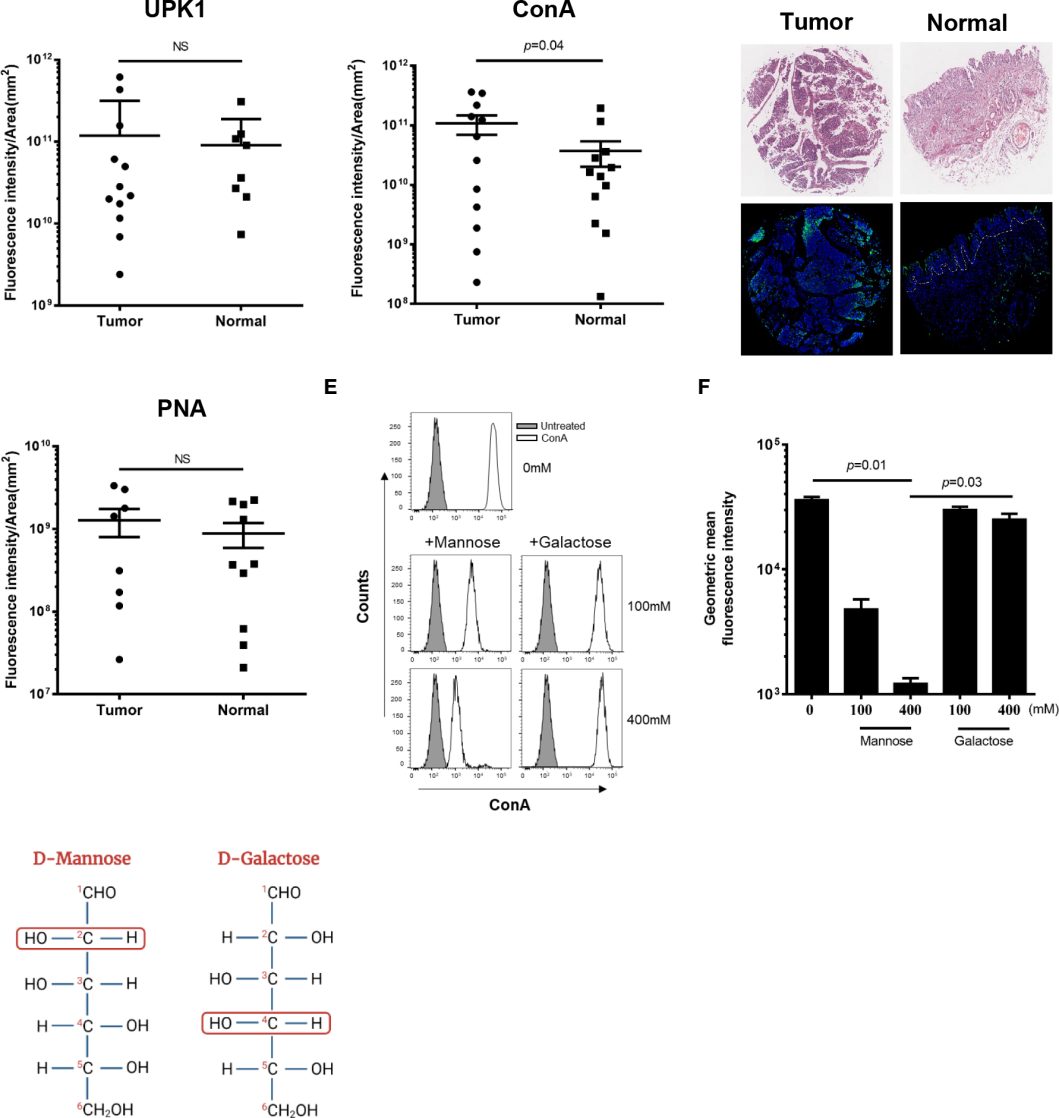
Bladder cancer cells express high levels of mannose. Binding of FITC-conjugated UPK1 (anti-uroplakin Ia antibody) **(A)**, FITC-conjugated ConA **(B)**, or FITC-conjugated PNA **(D)** to normal and tumor human bladder cancer samples (TMA BL244a). Each symbol represents one core. Circular symbols represent cancer samples; rectangular symbols represent normal adjacent tissues. Fluorescence intensity was analysed under the same conditions and divided by region of interest (ROI) area (mm^2^). NS, non-significant (*p*>0.05). Error bars indicate mean with SD. *p* was calculated using two-tailed Wilcoxon matched-pairs signed rank test. **(C)** Images of representative stained TMA of human bladder cancer (Tumor) and normal tissue (Normal). Top panel: H&E staining. Bottom panel: Staining with FITC-conjugated mannose-specific ConA (green) and Hoechst dye (blue). Dashed lines indicate the epithelial-muscle border (ROI) in the normal bladder tissue. **(E)** Representative flow cytometry histograms of binding off FITC-conjugated ConA to MB49 cells. ConA staining were measured in the absence (upper panel) or presence of mannose (left, middle and bottom panels) or galactose (right, middle and bottom panels) in the two indicated concentrations (100 mM and 400 mM). Grey filled histograms represent untreated MB49 cells. **(F)** Flow cytometry analysis of attachment of FITC-conjugated ConA (160 nM) to the MB49 murine bladder cancer cell line in the absence (first left column) or presence of the indicated concentrations of mannose or galactose. Mean ± SEM of six independent experiments performed in triplicate are presented. *p* was calculated using Bonferroni corrected one-tailed Wilcoxon matched-pairs signed rank test. **(G)** Schematic illustration representing the configuration differences between the epimers D-Mannose (on the left) and D-Galactose (on the right). The second (in D-Mannose) and fourth (in D- Galactose) carbon groups are surrounded by red unfilled circles to emphasize the differences.

To compare mannose level in a large number of cancer and normal bladder samples under identical conditions, we used the BL244a human tissue microarray (TMA). This TMA contains samples of 12 bladder tumors and their matching normal tissues. Duplicate slides from this TMA were screened; one for mannose levels using a fluorescently conjugated, mannose-specific ConA lectin, and one for the level of uroplakin Ia, the mannose-enriched bladder glycoprotein, using fluorescently conjugated anti-uroplakin Ia. Uroplakin Ia levels in bladder cancer samples were found to be similar to those in the matching normal bladder control tissues (**Figure 1A**). In contrast, higher mannose levels were detected in bladder cancer samples compared to the matching normal bladder uroepithel (**Figure 1B**), as represented by the intense fluorescent staining shown in **Figure 1C**.

It was previously reported that Gal-GalNAc level is elevated in certain cancers (Abed et al., 2017), but increase of Gal-GalNAc was not detected in bladder cancer (Neal et al., 1987; Abed et al., 2017). To confirm these results, and quantify Gal-GalNAc level in bladder cancer, the BL244a TMA was stained with fluorescently labeled PNA, a Gal-GalNAc specific lectin. Unlike mannose, no significant differences in Gal-GalNAc levels could be detected between tumor and normal bladder tissue (**Figure 1D**).

We next used flow cytometry and competition assays to verify mannose expression in bladder cancer cells. We measured attachment of FITC-conjugated ConA to the MB49 mouse bladder cancer cell line in the presence or absence of two different concentrations of soluble mannose, or galactose used as control (**Figures 1E, F**). In agreement with previous results (Huo et al., 2022), we detected ConA binding to the MB49 cells (**Figures 1E, F**). Importantly, ConA attachment to the MB49 cells was inhibited by mannose in a statistically significant, dose-dependent manner (**Figure 1F**). Despite the structural similarity between mannose and galactose (**Figure 1G**), we were unable to detect any changes in ConA attachment to MB49 cells when galactose was used as a competitor (**Figures 1E, F**). Taken together, these results support the early ones (Huo et al., 2022) reporting that mannose is overexpressed in the surface of bladder tumors of humans and mice.

### High mannose levels enable UPEC to preferentially bind bladder cancer cells in a type 1 fimbria dependent, mannose inhibitable mechanism

**Figure 2:**
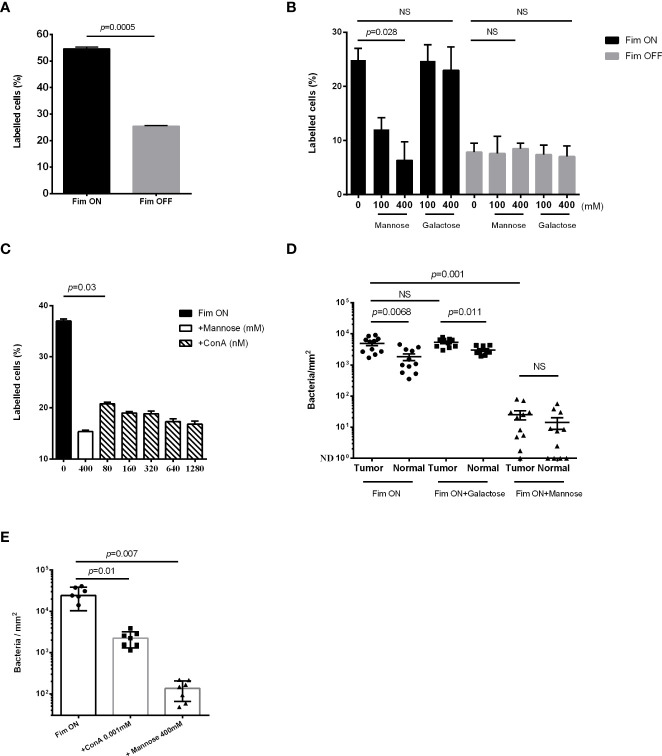
High mannose levels increase type 1 fimbria mediated, UPEC attachment to bladder cancer. **(A, B, C)** Flow cytometry analysis of GFP-expressing UPEC Fim ON or Fim OFF attachment to mouse bladder cancer cell lines MBT2 **(A)**, and MB49 **(B, C)**, in the absence **(A)** or presence **(B, C)** of the competing agents mannose, galactose, or ConA at the indicated concentrations. Data represents mean ± SEM of three independent experiments performed in triplicate in A and B, and five independent experiments performed in triplicate in C, NS, non-significant. *p* was calculated using two-tailed paired t-test (ratios between paired values) in A, Bonferroni corrected two-tailed paired t-test (differences between paired values) in B, and one-tailed Wilcoxon matched-pairs signed rank test in C. **(D, E)** Fluorescence microscopy analysis of the attachment of GFP-expressing Fim ON to bladder cancer samples (Tumor) and normal adjacent tissue (Normal) (BL244 tissue microarray) **(D)**, and to bladder cancer samples (T124a tissue microarray) **(E)**, in the absence or presence of 400 mM mannose, 400 mM galactose or 0.001 mM ConA. Quantitation of bacteria was performed as described in materials and methods and then divided by ROI areas. NS, non-significant (p>0.05). Each symbol represents one core. Means are shown with SD. *p* was calculated using Bonferroni corrected two-tailed Wilcoxon matched-pairs signed rank test in **(D)**, and Bonferroni corrected one-tailed Wilcoxon matched-pairs signed rank test in **(E)**.

The type 1 fimbria of uropathogenic *E. coli* (UPEC) binds mannose residues present on uroplakin Ia in the uroepithelium (Zhou et al., 2001). In particular, the mannose-binding adhesin FimH at the tip of the fimbria, mediates this bacterial-mannose interlinkage (Subashchandrabose and Mobley, 2015). In accordance with the results showing higher mannose levels in bladder cancer cells compared to non-cancerous ones (**Figures 1B, C**), we expected an increased attachment of type 1 fimbria - expressing bacteria to bladder cancer cells. To investigate this, we compared attachment of the GFP-expressing Fim ON and Fim OFF mutants of UPEC CFT073 (see Materials and Methods) to bladder cancer cell lines. Flow cytometry analysis of the attachment of the fluorescent bacteria to the MBT2 (**Figure 2A**) and MB49 (**Figure 2B**) mouse bladder cancer cell lines, revealed that type 1 fimbria plays a key role in UPEC attachment to mouse bladder cancer cells (**Figures 2A, B**). This type 1 fimbria mediated attachment is mannose sensitive and unaffected by galactose used as control (**Figure 2B**). Lastly, we tested whether the mannose lectin ConA, can compete with the attachment of GFP-expressing Fim ON to MB49. As expected, ConA (similar to mannose used as control), impaired the attachment of Fim ON to the bladder cancer cells (**Figure 2C**).

Due to its mannose-specific binding (McLellan et al., 2021; Mohammad Zadeh et al., 2021), we used type 1 fimbria expressing UPEC to confirm higher mannose levels in human bladder cancer tissue, compared to non-cancerous bladder uroepithelium. Attachment of GFP-expressing Fim ON to human tumor or normal bladder tissue was monitored in the absence or presence of soluble mannose, galactose (**Figure 2D**), or ConA (**Figure 2E**). Fluorescence-microscopy analysis demonstrated a significantly higher attachment of Fim ON to bladder cancer tissue, compared to normal bladder surfaces. Mannose, as opposed to galactose used as control, significantly impaired Fim ON attachment to bladder tissues (both normal and cancer) and eliminated the increased binding of Fim ON to the cancer tissues (**Figure 2D**). ConA, the mannose lectin, also competed with Fim ON, and significantly reduced its binding to bladder cancer tissues (similar to mannose used as control) (**Figure 2E**).

These data indicate that mannose is overexpressed in human and mouse bladder cancer, and that type 1 fimbriated UPEC binds to bladder cancer cells in a preferred manner compared to normal bladder tissue.

## Discussion

The majority of bladder cancers are transitional cell carcinomas (TCC) (Metts et al., 2000), and in most cases (75%), the tumor cells do not invade the underlying muscle tissue, and the tumor is defined as NMIBC (Tomaszewski and Smaldone, 2010). Following standard cystoscopy resection, high recurrence-risk patients receive adjuvant BCG immunotherapy (Grange et al., 2008). Although, BCG treatment has indisputable effectiveness in decreasing recurrences, it induces problematic side effects and provides no therapeutic benefit in some patients (Miyazaki et al., 2013; Brausi et al., 2014). Thus, efforts are invested to develop new adjuvant therapies which can replace, or be used alongside, BCG treatment. These include lectin-drug conjugates and bacterial-based immunotherapies. The ConA-epirubicin conjugate (Huo et al., 2022), and the adjuvant immunotherapies PA-MSHA (Wang et al., 2021) and rBCG-S.FimH (Zhang et al., 2022), were all shown to be more effective than BCG, against bladder cancer in the mouse orthotropic model.

Our study supports previous reports (Takayama, 1984; Neal et al., 1987; Huo et al., 2022) that using ConA, found that mannose is overexpressed in bladder cancer cells compared to normal uroepithelium. Increased mannosylation in bladder cancer was also confirmed here by mannose-sensitive, increased attachment of type 1 fimbria -expressing UPEC. Type 1 fimbria expressed in *E. coli* for which the primary sugar specificity is D-mannose, is expressed on the surface of many members of the Enterobacteriaceae. In agreement with our results, FimH of *Salmonella* Enteritidis and of *Salmonella* Typhimurium were shown to mediate their attachment to the bladder cancer cell line Hu 1703He (Kisiela et al., 2006).

In conclusion, our results demonstrate hypermannosylation in bladder cancer, and indicate that tumor-increased mannosylation mediates the advantage of the ConA-epirubicin conjugate, PA-MSHA, and rBCG-S.FimH treatments over BCG treatment in the mouse model of bladder cancer. Our results also suggest that the UPEC CFT073 Fim ON mutant that expresses high levels of mannose – binding, type 1 fimbria; and type 1 fimbria -expressing *Salmonella* Typhimurium [one of the bacterial species most studied as a bioengineered bacterium for cancer therapy (Chen et al., 2022)], have potential for use as vehicles for specific targeting of bladder cancer.

Our study bears some limitations: Although our study shows that mannose level is higher in human bladder cancer tissue compared to normal adjacent tissue, the mouse cell lines used in this study all derived from bladder cancer cells preventing the ability to compare cancer mannose level with that of normal mouse uroepithelium. In addition, while we show that type 1 fimbria mediates UPEC attachment to hypermannosylated bladder cancer tissue, in this study we do not focus on the specific involvement of FimH in this interaction.

## Data availability statement

The original contributions presented in the study are included in the article/supplementary material. Further inquiries can be directed to the corresponding authors.

## Author contributions

NM designed, carried out experiments and participated in writing the ms. VS carried out experiments, participated in writing the ms. CG, VY, OM, GB, designed experiments, and participated in writing ms. All authors contributed to the article and approved the submitted version.

## Funding

This work was supported by the Israel Cancer Research Fund Project grant, and the Israel Science Foundation Moked grant.

## Acknowledgments

We thank Dr. Asaf Sol for his valuable help in generating the figures.

## Conflict of interest

CG, VY, OM and GB are the inventors on a patent application (No. 10,918,677 B2: entitled “Attenuated or inactivated pathogenic *Escherichia coli* for treating urogenital cancer”) by the Hebrew University of Jerusalem, and the Hadassah Medical Organization.

The remaining authors declare that the research was conducted in the absence of any commercial or financial relationships that could be construed as a potential conflict of interest.

## Publisher’s note

All claims expressed in this article are solely those of the authors and do not necessarily represent those of their affiliated organizations, or those of the publisher, the editors and the reviewers. Any product that may be evaluated in this article, or claim that may be made by its manufacturer, is not guaranteed or endorsed by the publisher.
